# Controlling dispersity in aqueous atom transfer radical polymerization: rapid and quantitative synthesis of one-pot block copolymers†

**DOI:** 10.1039/d1sc04241f

**Published:** 2021-09-23

**Authors:** Hyun Suk Wang, Kostas Parkatzidis, Simon Harrisson, Nghia P. Truong, Athina Anastasaki

**Affiliations:** Laboratory of Polymeric Materials, Department of Materials, ETH Zurich Vladimir-Prelog-Weg 5 Zurich Switzerland nghia.truong@mat.ethz.ch athina.anastasaki@mat.ethz.ch; LCPO, ENSCBP/CNRS/Université de Bordeaux, UMR5629 Pessac France

## Abstract

The dispersity (*Đ*) of a polymer is a key parameter in material design, and variations in *Đ* can have a strong influence on fundamental polymer properties. Despite its importance, current polymerization strategies to control *Đ* operate exclusively in organic media and are limited by slow polymerization rates, moderate conversions, significant loss of initiator efficiency and lack of dispersity control in block copolymers. Here, we demonstrate a rapid and quantitative method to tailor *Đ* of both homo and block copolymers in aqueous atom transfer radical polymerization. By using excess ligand to regulate the dissociation of bromide ions from the copper deactivator complexes, a wide range of monomodal molecular weight distributions (1.08 < *Đ* < 1.60) can be obtained within 10 min while achieving very high monomer conversions (∼99%). Despite the high conversions and the broad molecular weight distributions, very high end-group fidelity is maintained as exemplified by the ability to synthesize *in situ* diblock copolymers with absolute control over the dispersity of either block (*e.g.* low *Đ* → high *Đ*, high *Đ* → high *Đ*, high *Đ* → low *Đ*). The potential of our approach is further highlighted by the synthesis of complex pentablock and decablock copolymers without any need for purification between the iterative block formation steps. Other benefits of our methodology include the possibility to control *Đ* without affecting the *M*_n_, the interesting mechanistic concept that sheds light onto aqueous polymerizations and the capability to operate in the presence of air.

## Abstract 2

The dispersity of polymers is efficiently controlled in aqueous atom transfer radical polymerization by modulating the reversible dissociation of the bromide ion from the copper deactivator.

## Graphical abstract

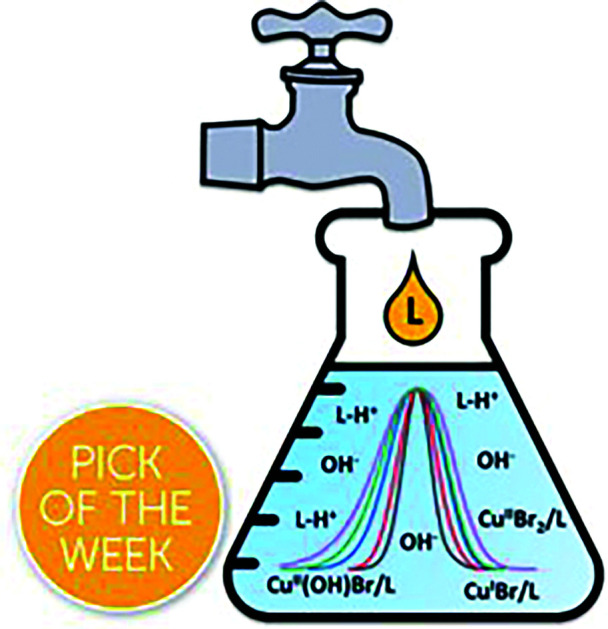

## Introduction

Synthetic polymers differ markedly from small molecules and biomacromolecules such as proteins in that they comprise a distribution of molecular weights. Such distribution, commonly quantified by dispersity (*Đ*), significantly influences a material's properties as it determines both the type and degree of inter- and intra-chain interactions of the constituent polymers.^1,2^ Reversible deactivation radical polymerization, also referred to as controlled radical polymerization,^3^ has traditionally focused on reducing *Đ* to the minimum, a strategy attributed to “gaining control” of the polymerization *via* diminishing chain termination and side reactions. As a consequence, high-dispersity polymers have often been considered undesirable and have widely become identified with “dead” polymer chains, *i.e.* chains that have lost their end-group and cannot undergo further chain extension. However, in many applications high-*Đ* polymers exhibit advantageous properties over their low-*Đ* counterparts including in melt rheology,^4^ electrospinning,^5^ nanoparticle brushes,^6^ and self-assembly.^7–9^ In fact, grades of industrially produced HDPE are characterized by their dispersities and have very different applications,^10^ suggesting that there is no “ideal” dispersity. In order to expand the potential applications of polymers consisting of variable molecular weight distributions, methodologies that can provide access to a wide range of *Đs* while exhibiting high end-group fidelity are required.

To this end, various groups have explored different avenues to control polymer *Đ*. At the early stages, molecular weight distributions were tailored by blending pre-synthesized polymer samples (either of low or high *Đ*) of different molecular weights. Junkers' and our group provided further insight by developing predictive frameworks that allowed custom design of molecular weight distributions.^11–15^ Recently, Fors and coworkers introduced an innovative semi-batch process to tune the dispersity by regulating initiation in nitroxide-mediated,^16^ anionic,^17,18^ and coordination polymerization.^19^ In a different approach, Goto and co-workers exploited the addition of a small amount of a co-monomer during the organocatalyzed reversible complexation-mediated polymerization, enabling access to various architectures and *Đs*.^20^ Matyjaszewski's and our group were independently able to demonstrate efficient tailoring of dispersity by reducing the catalyst concentration in atom transfer radical polymerization (ATRP).^21–24^ Other approaches include the use of photochromic initiators,^25^ mixtures of RAFT (reversible addition–fragmentation chain transfer) agents^26,27^ and initiators,^28^ flow chemistry,^29–35^ additives,^36^ and reducing/termination agents.^37,38^

However, these methods operate exclusively in organic media and, to date, no strategy has been reported that can control *Đ* in aqueous ATRP. This is a significant omission limiting both the polymer and application scope. This limitation is partly due to additional complications in aqueous polymerizations, especially for ATRP, which cause increased termination events and side reactions in the form of rapid end-group hydrolysis and dissociation of the deactivator.^39^ Another issue with existing methods is that the *Đ* of diblock copolymers cannot be fully controlled. For instance, in ATRP it is not currently possible to meaningfully increase the dispersity of a diblock copolymer upon chain-extension (*i.e.* low *Đ* first block → high *Đ* diblock). In a similar vein, when employing RAFT, the *Đ* of the diblock depends on the *Đ* of the initial homopolymer and the RAFT end group, and cannot be arbitrarily controlled using current methods. The ability to control the *Đ* in both blocks would be highly advantageous and a step forward in the field of controlled radical polymerization as it would provide access to well-defined dually disperse (in molecular weight and composition) polymeric materials. Furthermore, achieving high monomer conversions when targeting polymers of various *Đs* is very challenging due to increased termination events when monomer concentration is depleted and also because a significant deceleration of the polymerization rate is often observed when synthesizing such materials.^40^ For the same reason, one-pot (or “*in situ*”) diblock copolymers with tunable *Đ* have also not been reported. Last but not least, low initiator efficiency is typically observed for high *Đ* polymers,^22,41^ thus compromising the efficiency of a given system in block copolymer synthesis.

**Scheme 1:**
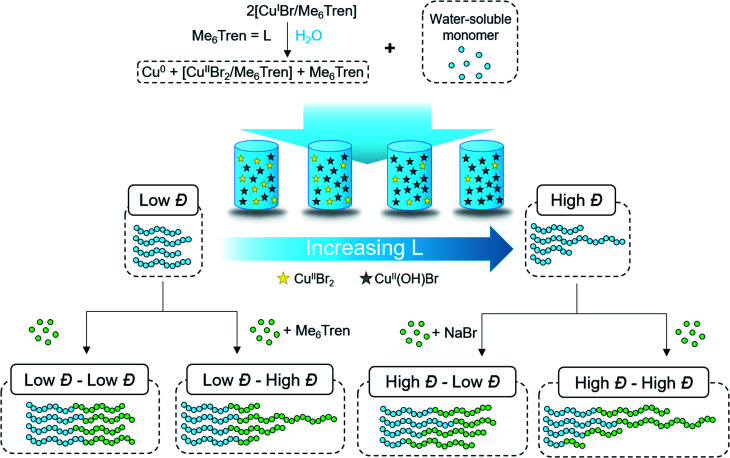
Aqueous approach to tailoring the dispersity of water-soluble polymers.

## Results & discussion

### Synthesis of water-soluble polymers with tunable dispersities

According to the well-established ATRP equation (eqn (1)), where *k*_p_ and *q* are the propagation constant and conversion, respectively, lowering the concentration of deactivator can lead to polymers with higher *Đs*. Thus, the key to successfully controlling polymer *Đ* in ATRP is to determine a way to regulate the deactivator concentration ([Cu^II^Br_2_]). In organic media this is possible by directly reducing [Cu^II^Br_2_]. However, this is particularly challenging in aqueous media as small amounts of copper deactivator are susceptible to significant halide dissociation.^42,43^ Hence, our direction was shifted to an alternative method (Scheme 1) whereby the *in situ* disproportionation of Cu^I^Br (yielding Cu^0^ and Cu^II^Br_2_) is exploited prior to the addition of the initiator and monomer.^44^ In particular, 2,3-dihydroxypropyl 2-bromo-2-methylpropanoate (GlyBiB) was used as the water-soluble ATRP initiator, tris(dimethylamino)ethyl amine (Me_6_Tren) as the ligand and *N*-isopropylacrylamide (NIPAM) as the monomer. The targeted degree of polymerization (DP) was set to 120. 2.4 equivalents of Cu^I^Br/Me_6_Tren (with respect to the initiator; [Cu^I^Br] : [Me_6_Tren] = [1] : [1]) were left to disproportionate in deionized water for 10 min, followed by the addition of an aqueous solution containing the initiator and monomer. Within 10 min, a polymer with fairly low dispersity (*Đ* ∼ 1.15) was obtained at quantitative conversion (>99%) (Table S1, Fig. S2†). We hypothesized that by reducing the initial [Cu^I^Br/Me_6_Tren], less [Cu^II^Br/Me_6_Tren] would be produced from *in situ* disproportionation, yielding polymers with higher *Đ*. Indeed, when 0.8 equivalents of [Cu^I^Br/Me_6_Tren] were employed, a higher *Đ* was attained (*Đ* ∼ 1.26) (Table S1, Fig. S4†). However, upon further decreasing [Cu^I^Br/Me_6_Tren] to 0.6 equivalents, a noticeable peak bimodality was observed (Fig. S5†), and below 0.4 equivalents complete loss of control over polymerization was observed (*Đ* ∼ 3.5), indicating insufficient deactivation under the conditions utilized (Table S1, Fig. S6–S8†). As such, simply lowering [Cu^I^Br/Me_6_Tren] proved ineffective and a different methodology was required to control polymer dispersity.1
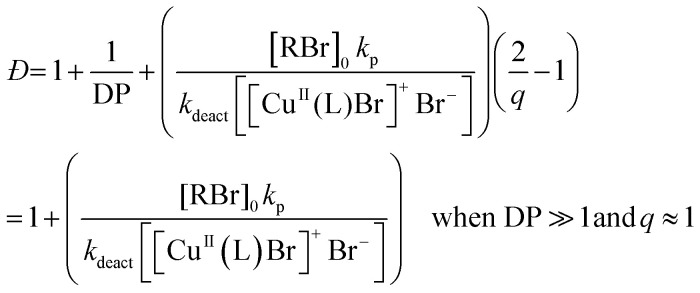


Instead, we envisioned that to tune polymer *Đ,* the amount of active copper deactivator complex [Cu^II^L(Br)]^+^Br^−^ (where L is the ligand Me_6_Tren) could be regulated *in situ* by exploiting its dissociation reaction in aqueous solution. In basic aqueous solution, OH^−^ ions could replace bromide of the active deactivator complex [Cu^II^L(Br)]^+^Br^−^, forming an inactive copper complex [Cu^II^L(OH)]^+^Br^−^. As such, we hypothesized that by modulating the Me_6_Tren ligand concentration, which is also a Lewis base that can react with water to produce OH^−^ ions, we could tune the amount of active copper deactivator ([Cu^II^L(Br)]^+^Br^−^) and ultimately control polymer *Đ* (see eqn (2)).

2 [Cu^II^L(Br)]^+^Br^−^ + L + H_2_O ⇌ [Cu^II^L(OH)]^+^Br^−^ + LH^+^ + Br^−^

**Fig. 1:**
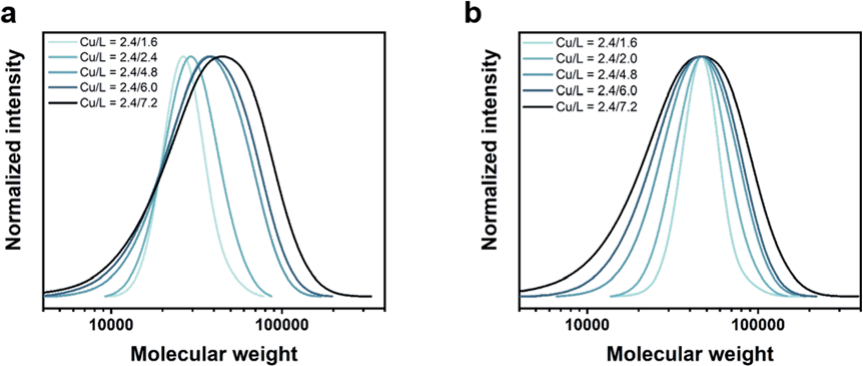
Tuning the dispersity of PNIPAM in water using different [Cu^I^Br] : [Me_6_Tren] ratios while maintaining constant (a) target DP and (b) *M*_p_.

To test this hypothesis, the homopolymerization of NIPAM was carried out with a sub-stoichiometric ligand concentration (with respect to Cu^I^Br) using the following conditions: [NIPAM] : [Initiator] : [Cu^I^Br] : [Me_6_Tren] = [120] : [1] : [2.4] : [1.6]. Within 6 min, the complete disappearance of the vinyl signals between 5.5 and 6.5 ppm in the ^1^H-NMR spectrum confirmed full monomer conversion (>99%) (Fig. S9†) and size exclusion chromatography (SEC) showed a symmetrical and monomodal molecular weight distribution with low dispersity (*Đ* ∼ 1.09, *M*_n_ ∼ 27 700) (Fig. 1a, Table S2†). When a ratio of ([Cu^I^Br] : [Me_6_Tren] = [2.4] : [2.4] was employed, a polymer with comparable molecular weight could be obtained, albeit with a slightly higher dispersity (*Đ* ∼ 1.14, *M*_n_ ∼ 28 400). Following our initial hypothesis, further increase in the ligand concentration ([Cu^I^Br] : [Me_6_Tren] = [2.4] : [4.8], [2.4] : [6.0], and [2.4] : [7.2]) led to a gradual broadening of the molecular weight distribution, yielding polymers with *Đ* ∼ 1.33, 1.40 and 1.60, respectively (Fig. 1a, Table S2†). Importantly, in all cases, monomodal molecular weight distributions could be maintained without any visible tailing, suggesting negligible termination. In addition, quantitative conversions (>98%) could be achieved within 10 min regardless of the targeted *Đ*. This is a significant improvement over previous methods in which moderate conversions (*e.g.* 16%) and slower rates of polymerization (*e.g.* 2–5 h) were observed for higher *Đ* polymers.^22^ Another major advantage of our strategy is that altering ligand concentration only affects *Đ* rather than *M*_n_. In previous systems, changing *Đ* was accompanied by a significant variation in *M*_n_.^22^ Alternatively, we were able to maintain a constant peak molecular weight *M*_p_ (and thereby, also the weight-average molecular weight *M*_w_) as the physical properties of polymeric materials hinge more critically on their *M*_w_ rather than *M*_n_ (Fig. 1b, Table S3†). The latter data also allow for a better visualization of the gradual broadening of the molecular weight distributions.

### Unravelling the effect of ligand on the dissociation of Cu^II^Br_2_

We were also interested in providing a preliminary mechanistic insight to further understand the effectiveness of our methodology. Our initial hypothesis was that the ligand could *in situ* reduce the concentration of active copper deactivator ([Cu^II^L(Br)]^+^Br) *via* the reaction shown in eqn (2), thus producing polymers with higher *Đ*. If this were the case, and following eqn (2), supplying bromide salts would drive the equilibrium back to the left-hand side and regenerate the active deactivator.^45,46^ To test this, NaBr was added to the polymerization with a threefold excess of ligand ([Cu^I^Br] : [Me_6_Tren] = [2.4] : [7.2]) which, without the salt, produced high-*Đ* PNIPAM. Indeed, when 120 eq. NaBr was added, the resulting *Đ* was 1.12, in stark contrast to reactions without NaBr that possessed a much higher dispersity (*Đ* = 1.6) (Fig. S10†). This data strongly suggest that dissociation is indeed the driving force behind the increase in *Đ*.

Next, to confirm that Me_6_Tren increased halide dissociation by producing hydroxyl ions, various amounts of NaOH were added to reactions with no excess ligand ([Cu^I^Br] : [Me_6_Tren] = [2.4] : [1.6]), which would also reduce the concentration of active copper deactivator and produce high-*Đ* PNIPAM (eqn (2)). In agreement with our hypothesis, increasing the NaOH content did indeed increase *Đ* as did excess ligands (Fig. S11, S14a and Table S4†). At low NaOH content (0.83 mM), the pH of the reaction was ∼10 and yielded *Đ* = 1.08. At a higher NaOH concentration (5.83 mM), the pH was ∼11, resulting in *Đ* = 1.70. The relationship between pH and halide dissociation can be qualitatively understood in terms of the generation of the inactive deactivator as shown in eqn (3). Qualitatively, the relationship between Me_6_Tren concentration and the ratio of inactive to active copper deactivator can be expressed with eqn (4), whose derivation can be found in the supporting information,† where *K*_*b*_ is the base dissociation constant of Me_6_Tren and *K*_d_ is the halide dissociation equilibrium constant for eqn (2).

3 [Cu^II^L(Br)]^+^Br^−^ + OH^−^ ⇌ [Cu^II^L(OH)]^+^Br^−^ + Br^−^

4
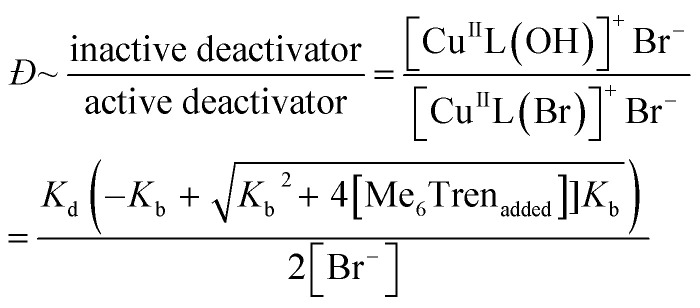


Although eqn (4) is not derived to quantitatively calculate the dispersity values, it can be seen that increasing ligand concentration increases *Đ* (Fig. S14b†) whereas an increase in [Br^−^] (directly correlated to the amount of NaBr added) decreases *Đ* (Fig. S10†), in agreement with our experimental results. Finally, eqn (4) can be further applied to the well-established equation for *Đ* in ATRP to give eqn (S6).† To summarize, we concluded that by increasing the ligand concentration, the concentration of the active [Cu^II^L(Br)]^+^Br^−^ deactivator was (reversibly) reduced through halide dissociation and thereby the rate of deactivation was decreased, ultimately leading to higher *Đ*s.

It is also worth mentioning that the role of excess ligand on the disproportionation is negligible in this case as full disproportionation was achieved prior to polymerization (Fig. S12 and S13†). Thus, the primary mechanism for the change in dispersity is attributed to halide dissociation rather than a lower generation of deactivator *via* incomplete disproportionation.

### Assessing chain-end fidelity in diblock copolymers with tunable *Đ*

**Fig. 2:**
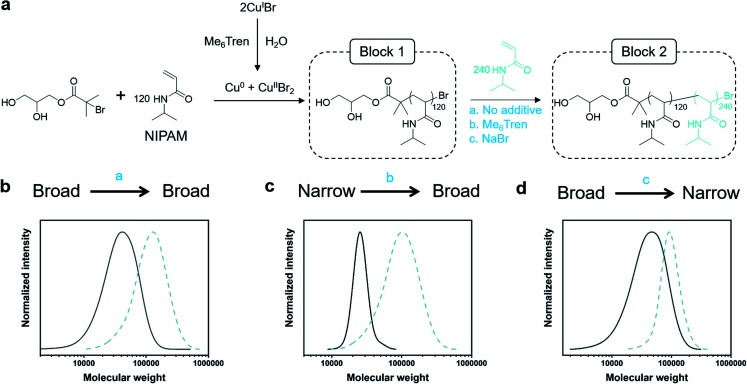
(a) Synthesis of one-pot PNIPAM-*b*-PNIPAM with individually tunable blocks *via* the Cu/L ratio for the first block and either Me_6_Tren or NaBr for the second block. (b) SEC traces for high-to-high, (c) low-to-high, and (d) high-to-low dispersity diblocks.

Dissociation is often considered to be an undesirable side reaction, yielding polymers with poor end-group fidelity. As we were interested in creating living high-*Đ* polymers, we aimed to explore the livingness of our polymers through the synthesis of a variety of diblock copolymers whereby any *Đ* combination would be feasible (Fig. 2a). In particular, three variations of chain extensions were designed: high-to-high, low-to-high and high-to-low *Đ*. In the first example, a high *Đ* PNIPAM (*Đ* ∼ 1.5, DP = 120) was prepared. After reaching full monomer conversion (>99%), a second aliquot of NIPAM was added, yielding a diblock copolymer with *Đ* ∼ 1.5 (Fig. 2b, Table S5†). It is worth noting that a high DP was targeted for the second block (DP 240) to visualize the clear shift in the SEC trace and thereby demonstrate the living nature of the high-*Đ* first block. As a demonstration of the versatility in terms of monomer scope, a high-to-high dispersity chain extension was performed with poly(hydroxyethyl acrylamide) (PHEAM) (Fig. S15†) yielding comparable data. For the low-to-high *Đ* block copolymer, a sub-stoichiometric amount of Me_6_Tren ([Cu^I^Br] : [Me_6_Tren] = [2.4] : [1.6]) was used for the synthesis of the first block to yield a PNIPAM with *Đ* = 1.07 and subsequently chain-extended with more monomer and an additional amount of Me_6_Tren (3.2 eq) to end up with a final *Đ* of 1.41 (Fig. 2c, Table S5†). To the best of our knowledge, this is the first demonstration of a living polymerization methodology that allows such low-to-high transition as previous methodologies – both (semi)batch and continuous systems – have limited control over the dispersity of the second block. Finally, for the high-to-low block copolymer, the first block was synthesized with threefold excess Me_6_Tren to Cu^I^Br ([Cu^I^Br] : [Me_6_Tren] = [2.4] : [7.2]) to yield a dispersity of 1.64 and subsequently chain-extended with a monomer solution (DP = 240) containing 120 eq. NaBr to yield quasi-diblock with a final dispersity of 1.17 (Fig. 2d, Table S5†). Identical data could also be obtained if instead of NaBr, we inject Cu^II^Br_2_ together with the monomer addition (Fig. S16†). Importantly, the livingness of this system is especially noteworthy given that a loss of the halogen at the chain end *via* hydrolysis is a notorious side reaction in water. In summary, these are the first examples of “*in situ*” diblocks with full control over the dispersity and extremely high monomer conversions for both blocks (>98%). Simply by adding either Me_6_Tren or NaBr (or, alternatively, Cu^II^Br_2_) to each chain extension, one could control the final *Đ* regardless of the *Đ* of the first block.

### Robustness and versatility of the methodology

**Fig. 3:**
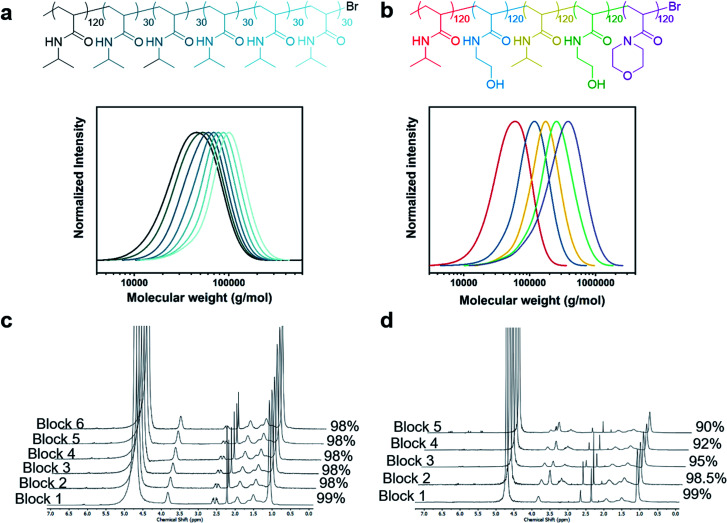
Chemical structure and SEC traces of (a) PNIPAM hexablock and (b) PNIPAM-*b*-PHEAM-*b*-PNIPAM-*b*-PHEAM-*b*-PNAM pentablock. Corresponding NMR spectra of each block in the (c) hexablock and (d) pentablock prior to monomer addition.

Considering the high polymerization speed, we were interested in investigating the reproducibility of our method. To achieve this, eight batches of high-*Đ* PNIPAM were polymerized under the same conditions ([Cu^I^Br] : [Me_6_Tren] = [2.4] : [7.2]) to quantitative conversion and their SEC traces were overlaid with each other (Fig. S17†). The deviations between batches were very minor, strongly evidencing the robustness and reproducibility of the methodology, especially since polymerization proceeds to full conversion, circumventing uncertainties regarding the duration of polymerization. In addition, we sought to explore whether we could extend our developed strategy to different monomer classes. Pleasingly, when poly(ethylene glycol) methyl ether acrylate (PEGA) was polymerized with different [Cu^I^Br] : [Me_6_Tren] ratios, a similar trend was observed, yielding materials with tunable *Đ*s (Fig. S18 and S19, Table S6 and S7†). We also attempted to tune the dispersity of the methacrylate equivalent, poly(ethylene glycol) methyl ether methacrylate, but a dispersity of 1.6 was obtained even in the absence of excess ligand (Fig. S20†). This indicated that the conditions currently employed are not suitable for obtaining a similar range of dispersities (*Đ* = 1.1–1.6). For methacrylates, electrochemical ATRP may offer a solution to obtain lower *Đ* (ref. 43,47–49) but this was deemed to be beyond the scope of the current work. To further probe the potential of our methodology, we were also interested in the limits of livingness for high-*Đ* systems as high *Đ* has traditionally been associated with chain termination. To this end we attempted to synthesize a high-*Đ* PNIPAM quasi-hexablock copolymer with a first block of target DP = 120 and the subsequent blocks of DP = 30 (Fig. 3). Each chain extension was allowed to proceed for 5–8 min to achieve quantitative conversions (>98%) prior to the subsequent chain extension (Fig. 3c). As seen in Fig. 3a, even with a first block of *Đ* = 1.55, there are clear shifts in the SEC trace upon each of the six chain extensions, indicating high retention of the terminal Br. A gradual increase in tailing on the lower molecular weight could be observed when additional chain extensions were attempted yielding a decablock copolymer (Fig. S21 and S22, Table S8†). For added chemical intricacy, we attempted to synthesize a challenging high-*Đ* pentablock copolymer consisting of PNIPAM, PHEAM, and poly(*N*-acryloylmorpholine) (PNAM) with a much higher total molecular weight. A DP of 120 was targeted for each block and clear shifts in the SEC traces despite the high dispersity and aqueous medium evidenced both the livingness of the system and its compatibility with other monomers (Fig. 3b). It is worth noting that the final *M*_n_ was as high as 221 600 g mol^−1^ which represents the highest molecular weight multiblock copolymer targeted to date (Table S9†). Moreover, it should be highlighted that such high-order multiblock copolymers can be synthesized in less than one hour for the decablock (∼90 min for the higher molecular weight pentablock copolymer) which also represents a remarkable acceleration in polymerization rate over previous ATRP methods.^50–52^ Last but not least, well-defined multiblock copolymers could be obtained in the absence of any external deoxygenation (Fig. S23, Table S10†) thus further highlighting the robustness of our approach and expanding the accessibility of our materials to non-experts.

## Conclusions

We introduce the first example of aqueous radical polymerization through which the dispersity can be efficiently tuned while maintaining monomodal molecular weight distributions. The key to our approach is to modulate the reversible dissociation of the bromide ion from copper deactivator by simply varying the ligand concentration, which ultimately determines the concentration of the active deactivator Cu^II^Br_2_ complex. The system is also compatible with other bases such as NaOH, but arguably the simplicity is maximized by increasing the content of an already-present component. Unlike previous methodologies, the dispersity could be fully controlled in both homopolymers and diblock copolymers while all reactions reach near-quantitative monomer conversions within 10 min. The very high end-group fidelity attained in our system was further exemplified by the rapid synthesis of *in situ* multiblock copolymers targeting both lower and higher molecular weight materials in an efficient manner. Both acrylamide- and acrylate-based monomers were compatible with our methodology, and high reproducibility and oxygen tolerance were also demonstrated. Given the tremendous importance of dispersity across different disciplines in both academia and industry, we envision this work will attract broad interest beyond the field of polymer chemistry.

## Data availability

General synthetic procedures, ^1^H-NMR, SEC, and UV-vis data supporting this article have been uploaded as part of the ESI.†

## Author contributions

A. A. and N.·P. T. conceived and designed the research. H. S. W. performed the experiments. K. P. contributed to the experiments. H. S. W., K.·P., S.·H., N.·P. T., A. A. contributed to the analysis of data and co-wrote the manuscript.

## Conflicts of interest

There are no conflicts to declare.

## Supplementary Material

SC-012-D1SC04241F-s001
