# Supplementary material for: Controlling dispersity in aqueous atom transfer radical polymerization: rapid and quantitative synthesis of one-pot block copolymers
Source: Chem Sci. 2021 Sep 23;12(43):14376–82. doi: 10.1039/d1sc04241f (PMC8580105; doi:10.1039/d1sc04241f)
Supplement: SC-012-D1SC04241F-s001 [file SC-012-D1SC04241F-s001.pdf]

Electronic Supplementary Material (ESI) for Chemical Science.
This journal is © The Royal Society of Chemistry 2021

Supplementary Information

Controlling Dispersity in Aqueous Atom Transfer Radical Polymerization: Rapid and Quantitative Synthesis of One-Pot Block Copolymers

Hyun Suk Wang,[a] Kostas Parkatzidis,[a] Simon Harrisson,[b] Nghia P. Truong,[a]* Athina Anastasaki[a]*

[a]Laboratory of Polymeric Materials, Department of Materials, ETH Zurich, Vladimir-Prelog-Weg 5, Zurich, Switzerland

[b]LCPO, ENSCBP/CNRS/Université de Bordeaux, UMR5629, Pessac, France

*email: nghia.truong@mat.ethz.ch; athina.anastasaki@mat.ethz.ch

Methods

Materials. NIPAM (Tokyo Chemical Industry, >98%), HEAM (Sigma-Aldrich, 97%), NAM (Sigma-Aldrich, 97%), PEGA (average M_n 480, Sigma-Aldrich), NaBr (Sigma-Aldrich, >99%), $CuBr_2$ (99%, Sigma-Aldrich), methyl acrylate (MA, Sigma-Aldrich, 99%), 2-cyano-2-propyl dodecyl trithiocarbonate (Sigma-Aldrich, 97%) and centrally deionized water were used without further purification. Me_6Tren was synthesized according to previous literature[46] and distilled prior to use. CuBr (Sigma-Aldrich, 98%) was washed sequentially with glacial acetic acid and ethanol numerous times and dried under vacuum. Water-soluble initiator 2, 3-dihydroxypropyl 2-bromo-2-methylpropanoate (GlyBiB) was synthesized according to the literature.[47]

NMR spectroscopy. ^{1}H-NMR spectra were recorded on a Bruker Avance-300 spectrometer using D_2O for samples from aqueous polymerizations and dimethyl sulfoxide-d_6 for samples from polymerizations in dimethyl sulfoxide. Chemical shifts are given in ppm downfield from tetramethylsilane and referenced to residual solvent proton signals.

Size-exclusion chromatography (SEC). SEC was measured on a Shimadzu equipment comprising a CBM-20A system controller, LC-20AD pump, SIL-20A automatic injector, 10.0 μm bead-size guard column (50 x 7.5 mm) followed by three KF-805L columns (300 x 8 mm, bead size: 10 μm, pore size maximum: 5000 Å), SPD-20A ultraviolet detector, and an RID-20A differential refractive index detector. The column temperature was maintained at 40 ℃ using a CTO-20A oven. The flow rate was set to 1 ml/min and with *N*, *N*-dimethylacetamide (DMAc, Acros, HPLC grade, with 0.03 w/v LiBr) as the eluent. Molecular weights were determined relative poly(methyl methacrylate) standards with molecular weights ranging from 5,000 to 1.5 x 10^6 g/mol (Agilent Technologies). All SEC samples were dissolved in DMAc and passed through 0.45 μm filters prior to analysis.

Polymerization of PNIPAM. To a vial fitted with a magnetic stir bar and a rubber septum, H_2O (3 mL) and varying amounts of Me_6TREN (depending on the target dispersity) were charged and the mixture was bubbled with nitrogen for 10 min. CuBr (12.7 mg, 0.0884 mmol) was then carefully added under continuous nitrogen bubbling. The nitrogen bubbling was left to proceed for another 10 min and then the blue suspension with purple red color copper (0) powder was allowed to stir at ambient temperature. At the same time, to another vial fitted with a rubber septum, H_2O (3 mL), 2, 3-dihydroxypropyl 2-bromo-2-methylpropanoate (8.9 mg, 0.0368 mmol) and NIPAM (0.5 g, 4.42 mmol) were charged and the mixture was bubbled with nitrogen for 15 min. Subsequently, the degassed monomer/initiator aqueous solution was transferred via a degassed syringe to the vial with Cu (0)/$CuBr_2$/Me_6TREN catalyst. The solution was allowed to polymerize at 0 °C for 6-10 min.

Polymerization of high-to-high dispersity PNIPAM diblock copolymer.

To a vial fitted with a magnetic stir bar and a rubber septum, H_2O (3 mL) and Me_6TREN (70.9 μL, 0.2651 mmol) were charged and the mixture was bubbled with nitrogen for 10 min. CuBr (12.7 mg, 0.0884 mmol) was then carefully added under continuous nitrogen bubbling. The nitrogen bubbling was left to proceed for another 10 min and then the blue suspension with purple red color copper (0) powder was allowed to stir at ambient temperature. At the same time, to another vial fitted with a rubber septum, H_2O (3 mL), 2, 3-dihydroxypropyl 2-bromo-2-methylpropanoate (8.9 mg, 0.0368 mmol) and NIPAM (0.5 g, 4.42 mmol) were charged and the mixture was bubbled with nitrogen for 15 min. Subsequently, the degassed monomer/initiator aqueous solution was transferred via a degassed syringe to the vial with Cu (0)/$CuBr_2$/Me_6TREN catalyst to initiate

polymerization. The solution was allowed to polymerize at 0 °C for 6 min. Subsequently, an aliquot of degassed NIPAM (1 g, 8.83 mmol) in H_2O (6 mL) was injected via a degassed syringe and the polymerization was left to proceed for 15 min.

Polymerization of high-to-high dispersity PNIPAM-*block*-PHEAM diblock copolymer. To a vial fitted with a magnetic stir bar and a rubber septum, H_2O (3 mL) and Me_6TREN (70.9 μL, 0.2651 mmol) were charged and the mixture was bubbled with nitrogen for 10 min. CuBr (12.7 mg, 0.0884 mmol) was then carefully added under continuous nitrogen bubbling. The nitrogen bubbling was left to proceed for another 10 min and then the blue suspension with purple red color copper (0) powder was allowed to stir at ambient temperature. At the same time, to another vial fitted with a rubber septum, H_2O (3 mL), 2, 3-dihydroxypropyl 2-bromo-2-methylpropanoate (8.9 mg, 0.0368 mmol) and NIPAM (0.5 g, 4.42 mmol) were charged and the mixture was bubbled with nitrogen for 15 min. Subsequently, the degassed monomer/initiator aqueous solution was transferred via a degassed syringe to the vial with Cu (0)/$CuBr_2$/Me_6TREN catalyst to initiate polymerization. The solution was allowed to polymerize at 0 °C for 6 min. Subsequently, an aliquot of degassed HEAM (1.02 g, 8.83 mmol) in H_2O (2.25 mL) was injected via a degassed syringe and the polymerization was left to proceed for 15 min.

Polymerization of high-to-low dispersity PNIPAM diblock copolymer. To a vial fitted with a magnetic stir bar and a rubber septum, H_2O (3 mL) and Me_6TREN (70.9 μL, 0.2651 mmol) were charged and the mixture was bubbled with nitrogen for 10 min. CuBr (12.7 mg, 0.0884 mmol) was then carefully added under continuous nitrogen bubbling. The nitrogen bubbling was left to proceed for another 10 min and then the blue suspension with purple red color copper (0) powder was allowed to stir at ambient temperature. At the same time, to another vial fitted with a rubber septum, H_2O (3 mL), 2, 3-dihydroxypropyl 2-bromo-2-methylpropanoate (8.9 mg, 0.0368 mmol) and NIPAM (0.5 g, 4.42 mmol) were charged and the mixture was bubbled with nitrogen for 15 min. Subsequently, the degassed monomer/initiator aqueous solution was transferred via a degassed syringe to the vial with Cu (0)/$CuBr_2$/Me_6TREN catalyst to initiate polymerization. The solution was allowed to polymerize at 0 °C for 6 min. Subsequently, an aliquot of degassed NIPAM (1 g, 8.83 mmol) and NaBr (0.455 g, 4.42 mmol) in H_2O (9 mL) was injected via a degassed syringe and the polymerization was left to proceed for 15 min.

Polymerization of low-to-high dispersity PNIPAM diblock copolymer. To a vial fitted with a magnetic stir bar and a rubber septum, H_2O (3 mL) and Me_6TREN (15.7 μL, 0.0589 mmol) were charged and the mixture was bubbled with nitrogen for 10 min. CuBr (12.7 mg, 0.0884 mmol) was then carefully added under continuous nitrogen bubbling. The nitrogen bubbling was left to proceed for another 10 min and then the blue suspension with purple red color copper (0) powder was allowed to stir at ambient temperature. At the same time, to another vial fitted with a rubber septum, H_2O (3 mL), 2, 3-dihydroxypropyl 2-bromo-2-methylpropanoate (8.9 mg, 0.0434 mmol) and NIPAM (0.5 g, 0.0368 mmol) were charged and the mixture was bubbled with nitrogen for 15 min. Subsequently, the degassed monomer/initiator aqueous solution was transferred via a degassed syringe to the vial with Cu (0)/$CuBr_2$/Me_6TREN catalyst to initiate polymerization. The solution was allowed to polymerize at 0 °C for 6 min. Subsequently, an aliquot of degassed NIPAM (1 g, 8.83 mmol) and Me_6Tren (31.5 μL, 0.0118 mmol) in H_2O (6 mL) was injected via a degassed syringe and the polymerization was left to proceed for 15 min.

Polymerization of PNIPAM in the presence of NaOH. A 0.1 M NaOH stock solution was first prepared. To a vial fitted with a magnetic stir bar and a rubber septum, H_2O (3 mL), Me_6TREN (15.7 μL, 0.0136 mmol), and various amounts of 0.1 M NaOH stock solution were charged and

the mixture was bubbled with nitrogen for 10 min. CuBr (12.7 mg, 0.0884 mmol) was then carefully added under continuous nitrogen bubbling. The nitrogen bubbling was left to proceed for another 15 min and then the blue suspension with purple red color copper (0) powder was allowed to stir at ambient temperature. At the same time, to another vial fitted with a rubber septum, H_2O (3 mL), 2, 3-dihydroxypropyl 2-bromo-2-methylpropanoate (8.9 mg, 0.0434 mmol) and NIPAM (0.5 g, 0.0368 mmol) were charged and the mixture was bubbled with nitrogen for 15 min.

Polymerization of in-situ high-dispersity PNIPAM decablock copolymer. To a vial fitted with a magnetic stir bar and a rubber septum, H_2O (3 mL) and Me_6TREN (70.9 μL, 0.2651 mmol) were charged and the mixture was bubbled with nitrogen for 10 min. CuBr (12.7 mg, 0.0884 mmol) was then carefully added under continuous nitrogen bubbling. The nitrogen bubbling was left to proceed for another 10 min and then the blue suspension with purple red color copper (0) powder was allowed to stir at ambient temperature. At the same time, to another vial fitted with a rubber septum, H_2O (3 mL), 2, 3-dihydroxypropyl 2-bromo-2-methylpropanoate (8.9 mg, 0.0368 mmol) and NIPAM (0.5 g, 4.42 mmol) were charged and the mixture was bubbled with nitrogen for 15 min. Subsequently, the degassed monomer/initiator aqueous solution was transferred via a degassed syringe to the vial with Cu (0)/$CuBr_2$/Me_6TREN catalyst to initiate polymerization. The solution was allowed to polymerize at 0 °C for 10 min. During polymerization, 9 aliquots of NIPAM (0.125 g, 1.10 mmol) solution in water (0.75 mL) were bubbled with nitrogen and injected subsequently to each other into the reaction after quantitative conversion of the previous block.

Polymerization of in-situ high-dispersity PNIPAM-*block*-PHEAM-*block*-PNIPAM-*block*-PHEAM-*block*-PNAM pentablock copolymer. To a vial fitted with a magnetic stir bar and a rubber septum, H_2O (1.5 mL) and Me_6TREN (35.4 μL, 0.1326 mmol) were charged and the mixture was bubbled with nitrogen for 10 min. CuBr (6.35 mg, 0.0442 mmol) was then carefully added under continuous nitrogen bubbling. The nitrogen bubbling was left to proceed for another 10 min and then the blue suspension with purple red color copper (0) powder was allowed to stir at ambient temperature. At the same time, to another vial fitted with a rubber septum, H_2O (1.5 mL), 2, 3-dihydroxypropyl 2-bromo-2-methylpropanoate (4.4 mg, 0.0184 mmol) and NIPAM (0.25 g, 2.21 mmol) were charged and the mixture was bubbled with nitrogen for 15 min. Subsequently, the degassed monomer/initiator aqueous solution was transferred via a degassed syringe to the vial with Cu (0)/$CuBr_2$/Me_6TREN catalyst to initiate polymerization. The solution was allowed to polymerize at 0 °C for 10 min. During polymerization, 1 aliquot of NIPAM (0.5 g, 4.42 mmol) solution in water (4.5 mL), 2 aliquots of HEAM (0.509 g, 4.42 mmol) solution in water (5 mL), and 1 aliquot of NAM (0.624 g, 4.42 mmol) in water (6 mL) were bubbled with nitrogen and injected subsequently to each other into the reaction after quantitative conversion of the previous block.

Polymerization of in-situ high-dispersity *block*-PHEAM-*block*-PNIPAM-*block*-PHEAM-*block*-PNAM pentablock copolymer in the presence of air. To a vial fitted with a magnetic stir bar and a rubber septum, H_2O (3 mL) and Me_6TREN (92.1 μL, 0.345 mmol) were charged. CuBr (16.5 mg, 0.115 mmol) was then added and stirred until a blue suspension with purple red color copper (0) powder formed. At the same time, to another vial fitted with a rubber septum, H_2O (4.5 mL), 2, 3-dihydroxypropyl 2-bromo-2-methylpropanoate (1.15 mg, 0.0479 mmol) and NIPAM (0.65 g, 5.74 mmol) were charged and the mixture was transferred via a non-degassed syringe to the vial with Cu (0)/$CuBr_2$/Me_6TREN catalyst to initiate polymerization. The solution was allowed to polymerize at 0 °C for 10 min. During polymerization, 1 aliquot of NIPAM (0.163 g, 1.44 mmol) solution in water (1 mL), 2 aliquots of HEAM (0.165 g, 1.44 mmol) solution in water (1 mL), and 1

aliquot of NAM (0.203 g, 1.44 mmol) in water (6 mL) were prepared and injected subsequently to each other into the reaction after quantitative conversion of the previous block.

Determination of monomer conversion. Monomer conversions were determined by NMR spectroscopy. For PNIPAM, integrals of the vinyl protons (δ ~ 5.6-6.1 ppm) from the monomer were compared to the integral of the –NCH- protons (δ ~ 3.7-4.0 ppm) from both the monomer and polymer. To calculate monomer conversion for individual PNIPAM blocks in quasi-block copolymers, the same calculation principle applies but –NCH- signals corresponding to previous blocks were subtracted prior to comparison with the vinyl integrals. For the pentablock copolymer, the same principles apply, only with different ppm shifts characteristic of the different monomers. -NCH_2- protons for PHEAM (δ ~ 3.4-3.7 ppm) and PNAM (δ ~ 3.6-3.8 ppm) were compared with their respective vinyl protons (δ ~ 5.6-6.3 ppm for PHEAM blocks, δ ~ 5.6-6.7 ppm for PNAM blocks) to determine monomer conversion.

Table S1. Effect of CuBr concentration on the dispersity of PNIPAM ([CuBr] : [Me_6Tren] = 1 : 1)

conversion (%)	[NIPAM]:[GlyBiB] :[CuBr]:[Me_6Tren]	M_n^{SEC} (Da)[a]	M_n^{theo} (Da)	$Đ$
60	[120] : [1] : [0.1] : [0.1]	364,400	8,400	3.00
99	[120] : [1] : [0.2] : [0.2]	23,600	13,700	4.86
99	[120] : [1] : [0.4] : [0.4]	25,200	13,700	3.54
99	[120] : [1] : [0.6] : [0.6]	25,400	13,700	1.84
99	[120] : [1] : [0.8] : [0.8]	29,600	13,700	1.26
99	[120] : [1] : [1.6] : [1.6]	29,400	13,700	1.15
99	[120] : [1] : [2.4] : [2.4]	29,900	13,700	1.15
99	[120] : [1] : [4.8] : [4.8]	29,600	13,700	1.13

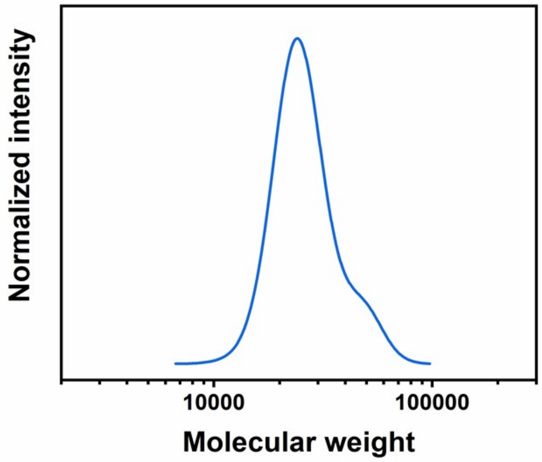

Figure S1. SEC traces of PNIPAM synthesized with [NIPAM] : [GlyBiB] : [CuBr] : [Me_6Tren] = [120] : [1] : [4.8]: [4.8].

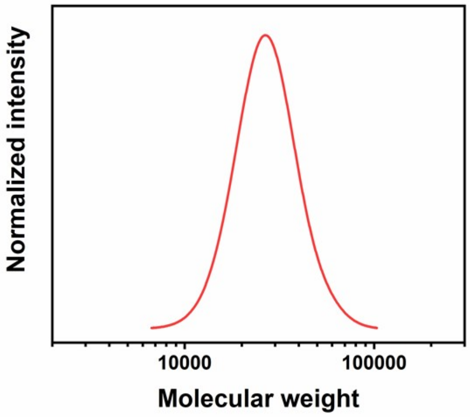

Figure S2. SEC traces of PNIPAM synthesized with [NIPAM] : [GlyBiB] : [CuBr] : [Me_6Tren] = [120] : [1] : [2.4] : [2.4].

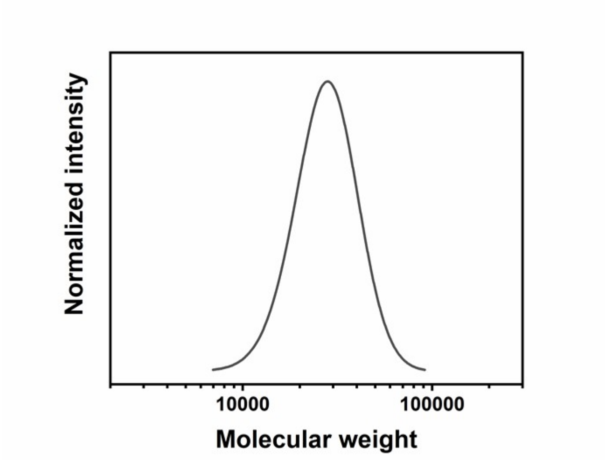

Figure S3. SEC traces of PNIPAM synthesized with [NIPAM] : [GlyBiB] : [CuBr] : [Me_6Tren] = [120] : [1] : [1.6] : [1.6].

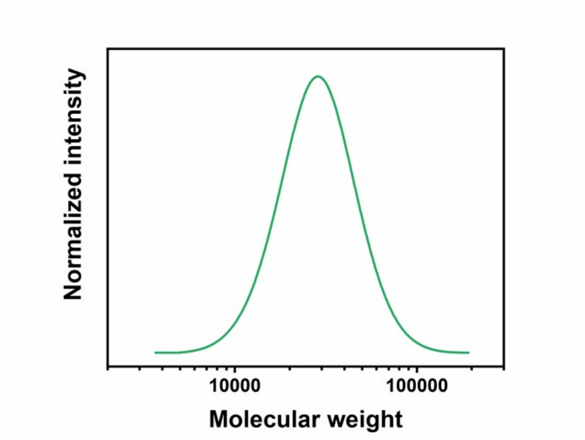

Figure S4. SEC traces of PNIPAM synthesized with [NIPAM] : [GlyBiB] : [CuBr] : [Me_6Tren] = [120] : [1] : [0.8] : [0.8].

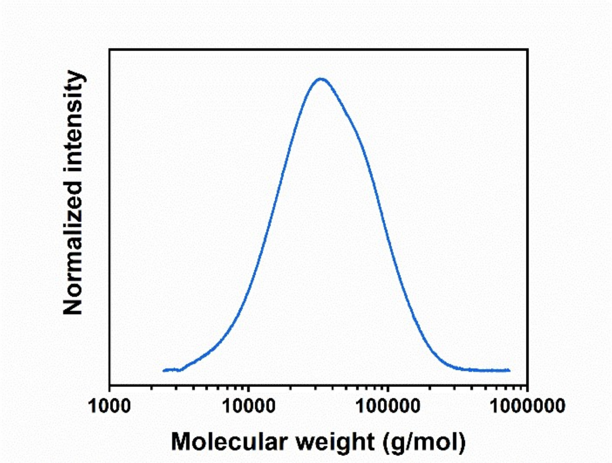

Figure S5. SEC traces of PNIPAM synthesized with [NIPAM] : [GlyBiB] : [CuBr] : [Me_6Tren] = [120] : [1] : [0.6] : [0.6].

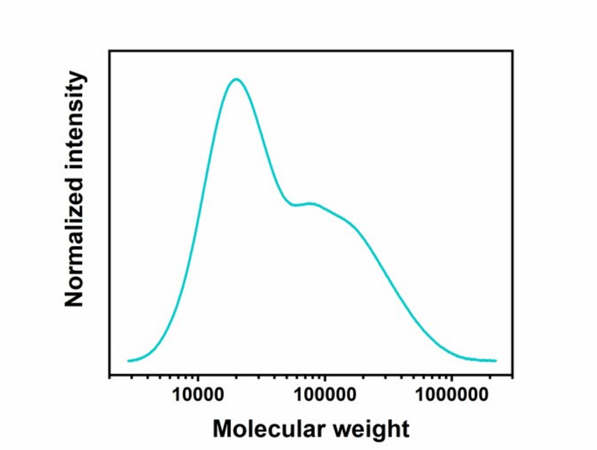

Figure S6. SEC traces of PNIPAM synthesized with [NIPAM] : [GlyBiB] : [CuBr] : [Me_6Tren] = [120] : [1] : [0.4] : [0.4].

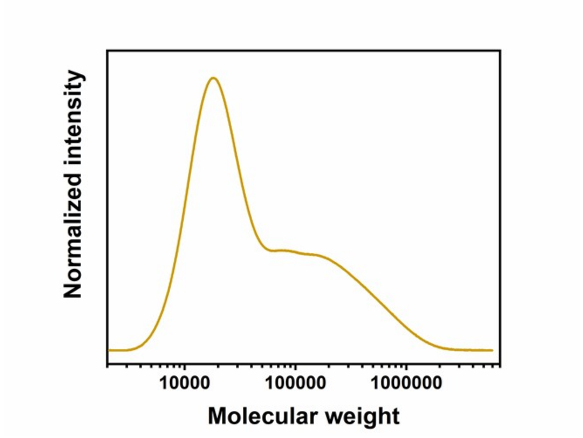

Figure S7. SEC traces of PNIPAM synthesized with [NIPAM] : [GlyBiB] : [CuBr] : [Me_6Tren] = [120] : [1] : [0.2] : [0.2].

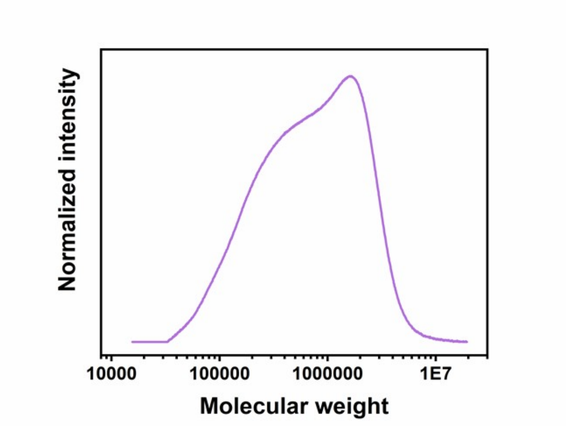

Figure S8. SEC traces of PNIPAM synthesized with [NIPAM] : [GlyBiB] : [CuBr] : [Me_6Tren] = [120] : [1] : [0.1] : [0.1].

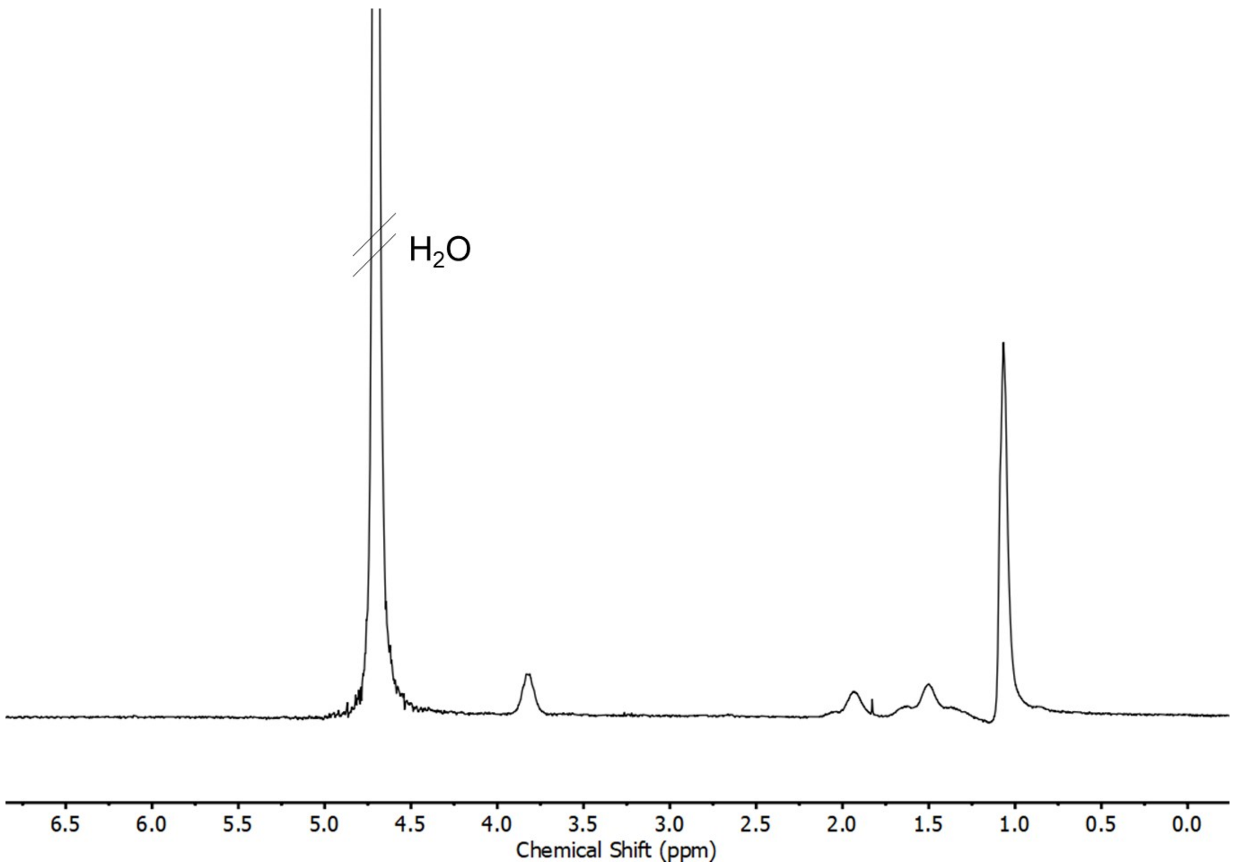

Figure S9. NMR spectrum of PNIPAM sampled after 6 min ([NIPAM] : [GlyBiB] : [CuBr] : [Me_6Tren] = [120] : [1] : [2.4] : [1.6]).

Table S2. Synthesis of PNIPAM using various [CuBr]: [Me_6Tren] ratios and maintaining constant target DP.

entry	conversion (%)	[NIPAM]:[GlyBiB]:[CuBr]:[Me_6Tren]	M_n^{SEC} (Da)[a]	M_n^{theo} (Da)	*Đ*
1	99	[120] : [1] : [2.4] : [1.6]	27,700	13,700	1.09
2	99	[120] : [1] : [2.4] : [2.4]	28,400	13,700	1.14
3	99	[120] : [1] : [2.4] : [4.8]	29,900	13,700	1.33
4	99	[120] : [1] : [2.4] : [6.0]	30,100	13,700	1.40
5	98	[120] : [1] : [2.4] : [7.2]	30,500	13,500	1.60

[a]the discrepancy between the theoretical and SEC-derived M_n is due to a combination of the different hydrodynamic volume of PNIPAM in the GPC eluent (*N*, *N*-dimethylacetamide) compared to the poly(methyl methacrylate) standards, and some apparent loss of bromide functionality from the GlyBiB initiator in water. To probe the effect of the end groups (bromine-less GlyBiB on one end, bromine on the other) on the SEC $M_{n,}$ PNIPAM was synthesized via RAFT polymerization using azobisisobutyronitrile as the initiator and analyzed by SEC (Table S11, Figure S24). The SEC M_n of RAFT-PNIPAM was 34% higher than the theoretical value, a

trend also found in the literature. Then, to test whether some dissociation of GlyBiB occurs in water, photo-ATRP of methyl acrylate (as poly(methyl acrylate) gives much more accurate values in SEC) was performed with GlyBiB in DMSO. Relatively good agreement (~14% difference) between theoretical and SEC M_n was found (Table S12, Figure S25), suggesting that the dissociation is much higher in water than organic solvent. Therefore, the discrepancy between the theoretical and SEC-M_n is a result of both the different hydrodynamic behavior compared with the PMMA polymer standard and loss of some bromide group from the initiator in water.

Table S3. Synthesis of PNIPAM using various [Cu]: [Me_6Tren] ratios and maintaining constant M_p

entry	conversion (%)	[NIPAM]:[GlyBiB]:[CuBr]:[Me_6Tren]	M_n^{SEC} (Da)	M_n^{theo} (Da)	M_p^{SEC}(Da)	*Đ*
1	96	[233] : [1] : [2.4] : [1.6]	44,500	25,600	46,500	1.08
2	99	[213] : [1] : [2.4] : [2.0]	43,500	24,100	46,900	1.13
3	99	[136] : [1] : [2.4] : [4.8]	38,500	15,500	45,800	1.26
4	99	[135] : [1] : [2.4] : [6.0]	34,600	15,400	46,900	1.36
5	93.5	[120] : [1] : [2.4] : [7.2]	29,200	12,900	46,900	1.64

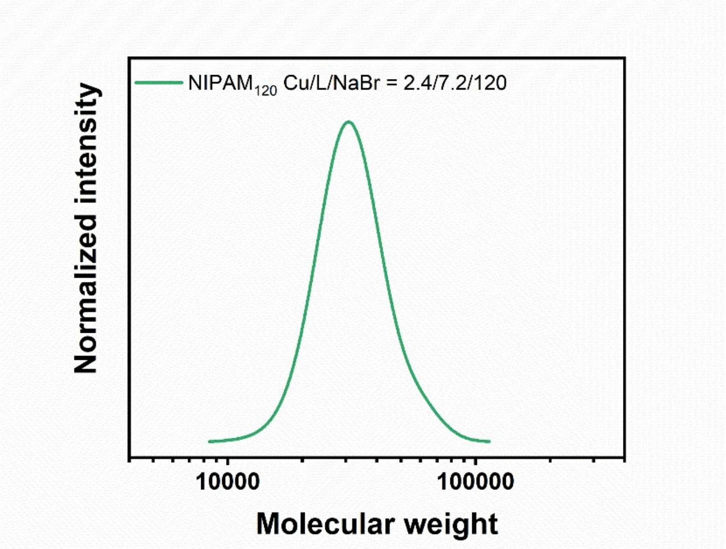

Figure S10. SEC trace of PNIPAM synthesized with high dispersity [CuBr] : [Me_6Tren] condition plus NaBr ([NIPAM] : [GlyBiB] : [CuBr] : [Me_6Tren] : [NaBr] = [120] : [1] : [2.4]: [7.2] : [120]).

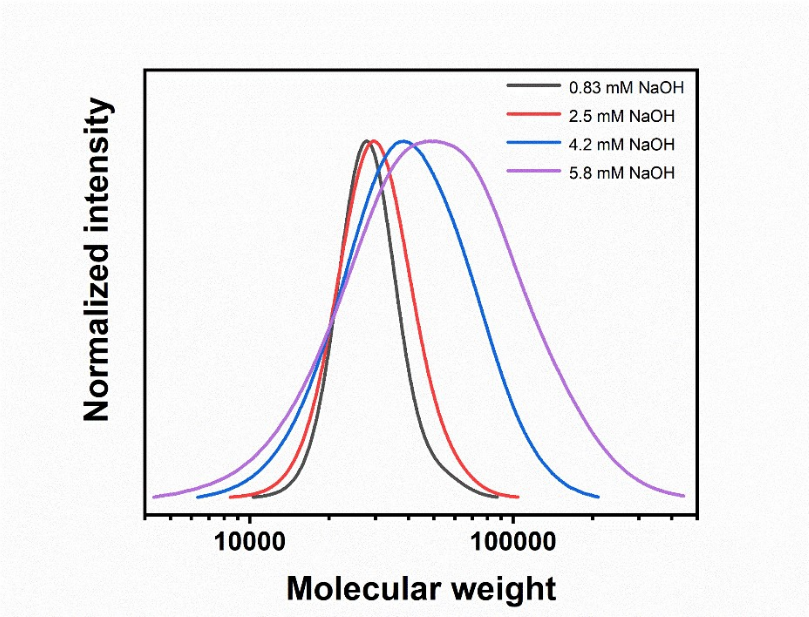

Figure S11. SEC traces of PNIPAM synthesized with low dispersity conditions ([NIPAM] : [GlyBiB] : [CuBr] : [Me_6Tren] = [120] : [1] : [2.4]: [1.6]) in the presence of various concentrations of NaOH. Dispersity values are 1.08 (black), 1.13 (red), 1.35 (blue), and 1.70 (purple).

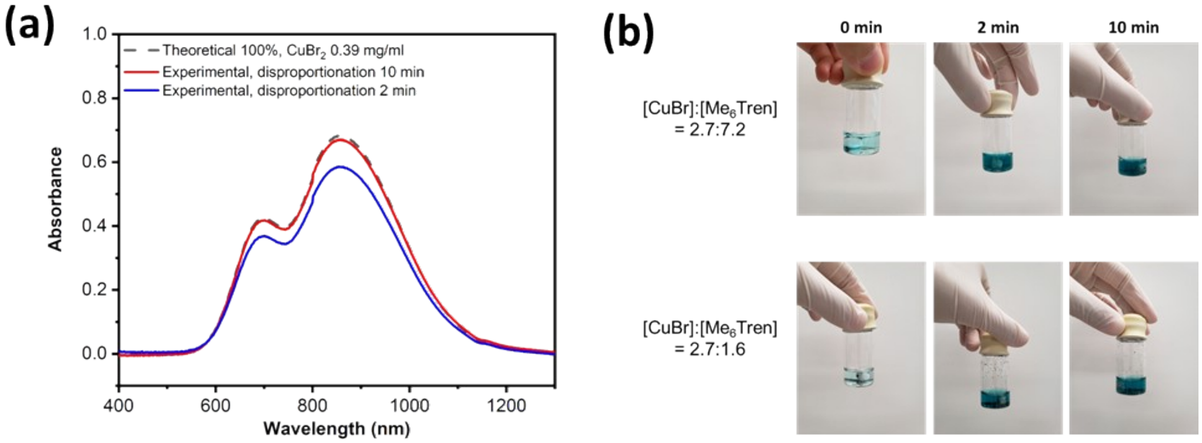

Figure S12. (a) UV-Vis spectra of CuBr disproportionation ([CuBr] : [Me6Tren] = [2.4] : [7.2]) in water at 2 min and 10 min. 86% and 99% disproportionation was achieved after 2 min and 10 min, respectively. The degree of disproportionation was calculated by comparing intensities of peak maxima at 860 nm. (b) Photos of the disproportionation after 0, 2, and 10 min.

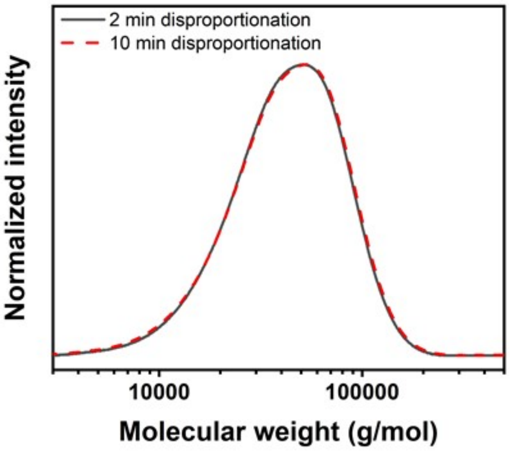

Figure S13. SEC traces of PNIPAM synthesized with high dispersity conditions ([NIPAM] : [GlyBiB] : [CuBr] : [Me_6Tren] = [120] : [1] : [2.4]: [7.2]) after different disproportionation times.

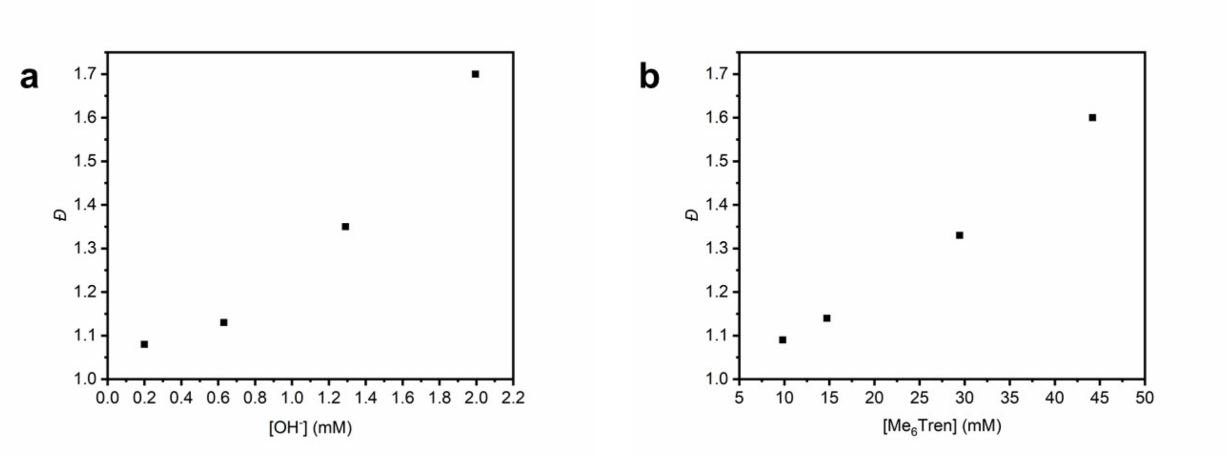

Figure S14. (a) Effect of [OH$^-$] on *Đ* for [NIPAM] : [GlyBiB] : [CuBr] : [Me$_6$Tren] : [NaOH] = [120] : [1] : [2.4] : [1.6] : [x]. The [OH$^-$] was calculated by measuring the pH of the solution after quantitative monomer conversion. (b) Effect of [Me$_6$Tren] on *Đ* for [NIPAM] : [GlyBiB] : [CuBr] : [Me$_6$Tren] = [120] : [1] : [2.4] : [x]. The points on the plot correspond to 1.6 eq, 2.4 eq, 4.8 eq, and 7.2 eq Me$_6$Tren and are converted to mM concentrations.

Table S4. Effect of [NaOH] on low-dispersity PNIPAM polymerization conditions.

[NIPAM]:[GlyBiB]:[CuBr]:[Me$_6$Tren]	[NaOH] (mM)	pH^a	[OH$^-$] (mM)^b	*Đ*	M_n^{SEC} (Da)	M_n^{theo} (Da)
[120] : [1] : [2.4] : [1.6]	0.83	10.3 ± 0.1	0.20	1.08	27,300	13,400
[120] : [1] : [2.4] : [1.6]	2.50	10.8 ± 0.1	0.63	1.13	28,200	13,400
[120] : [1] : [2.4] : [1.6]	4.17	11.1 ± 0.1	1.29	1.35	33,500	13,400
[120] : [1] : [2.4] : [1.6]	5.83	11.3 ± 0.2	2.00	1.70	37,200	13,400

Derivation of Eq 4.

We start with the Cu^{II} halide dissociation equilibrium

$[Cu^{II}L(Br)]^{+}Br^{-} + OH^{-} \rightleftharpoons [Cu^{II}L(OH)]^{+}Br^{-} + Br^{-}$ $\qquad K_d = \frac{[[Cu^{II}L(OH)]^{+}Br^{-}][Br^{-}]}{[[Cu^{II}L(Br)]^{+}Br^{-}][OH^{-}]}$

Rearranging the K_d equation, we get

$$\frac{[[Cu^{II}L(OH)]^{+}Br^{-}]}{[[Cu^{II}L(Br)]^{+}Br^{-}]} = \frac{K_d[OH^{-}]}{[Br^{-}]} \qquad \text{(S1)}$$

Meanwhile, we have the base equilibrium

$Me_6Tren + H_2O \rightleftharpoons Me_6Tren\text{-}H^{+} + OH^{-}$ $\qquad K_b = \frac{[Me_6Tren-H^{+}][OH^{-}]}{[Me_6Tren]}$

Setting

$[OH^{-}] = [Me_6Tren\text{-}H^{+}] = a$ and $[Me_6Tren_{added}] = C$, we get

$K_b = \frac{a \times a}{C - a}$ or $a^2 + aK_b - CK_b = 0$

Solving for a, we get

$$a = [OH^{-}] = \frac{-K_b \pm \sqrt{K_b^2 + 4CK_b}}{2} = \frac{-K_b + \sqrt{K_b^2 + 4[Me_6Tren_{added}]K_b}}{2} \qquad \text{(S2)}$$

Combining Eq S1 and S2, we get

$$\frac{[[Cu^{II}L(OH)]^{+}Br^{-}]}{[[Cu^{II}L(Br)]^{+}Br^{-}]} = \frac{K_d[OH^{-}]}{[Br^{-}]} = \frac{K_d(-K_b + \sqrt{K_b^2 + 4[Me_6Tren_{added}]K_b})}{2[Br^{-}]} \qquad \text{(4)}$$

Integration of Eq 4 into the ATRP equation.

Eq 4 can be expressed in terms of the Cu^{II} (disproportionated + added) concentration C_d as follows:

$$\frac{[[Cu^{II}L(OH)]^{+}Br^{-}]}{[[Cu^{II}L(Br)]^{+}Br^{-}]} = \frac{C_d - [[Cu^{II}L(Br)]^{+}Br^{-}]}{[[Cu^{II}L(Br)]^{+}Br^{-}]} = \frac{K_d(-K_b + \sqrt{K_b^2 + 4[Me_6Tren_{added}]K_b})}{2[Br^{-}]}$$

Rearranged,

$$\frac{1}{[[Cu^{II}L(Br)]^{+}Br^{-}]} = \frac{K_d(-K_b + \sqrt{K_b^2 + 4[Me_6Tren_{added}]K_b})}{2[Br^{-}]C_d} + \frac{1}{C_d} \qquad \text{(S3)}$$

Meanwhile, the ATRP equation for dispersity is expressed as

$$Đ = 1 + \frac{1}{DP} + \left(\frac{[RBr]_0 k_p}{k_{deact}[[Cu^{II}(L)Br]^+ Br^-]}\right)\left(\frac{2}{q} - 1\right)$$

$$= 1 + \left(\frac{[RBr]_0 k_p}{k_{deact}[[Cu^{II}(L)Br]^+ Br^-]}\right) \quad \text{when DP>>1 and } q \approx 1 \qquad (1)$$

Combining Eq S3 and Eq 1, we get

$$Đ = 1 + \left(\frac{[RBr]_0 k_p}{k_{deact}}\right)\left(\frac{K_d\left(-K_b + \sqrt{K_b^2 + 4[Me_6Tren_{added}]K_b}\right)}{2[Br^-]C_d} + \frac{1}{C_d}\right)$$

$$= 1 + \left(\frac{[RBr]_0 k_p}{k_{deact}C_d}\right)\left(\frac{K_d\left(-K_b + \sqrt{K_b^2 + 4[Me_6Tren_{added}]K_b}\right)}{2[Br^-]} + 1\right)$$

$$= 1 + \frac{[RBr]_0 k_p K_d\left(-K_b + \sqrt{K_b^2 + 4[Me_6Tren_{added}]K_b}\right)}{2k_{deact}C_d[Br^-]} + \frac{[RBr]_0 k_p}{k_{deact}C_d} \qquad (S6)$$

Table S5. Characterization of PNIPAM quasi-diblocks and calculation of theoretical dispersities.

Entry	(Block 1) M_n^{SEC} (Da)	(Block 1) $Đ$	(Diblock) M_n^{SEC} (Da)	(Diblock) $Đ$	(Block 2) $Đ_{expected}$[a]
Broad →Broad	29,000	1.54	91,000	1.48	1.95
Narrow → Broad	25,000	1.07	78,700	1.41	1.91
Broad → Narrow	29,200	1.64	87,000	1.17	1.22

Expected dispersities were calculated with the equation $Đ_{12} = w_1^2(Đ_1-1) + w_2^2(Đ_2-1) + 1$, where w_1 and w_2 denotes the weight fraction of block 1 and 2, respectively. As 120 eq and 240 eq of NIPAM were used for block 1 and 2, respectively, w_1 = 1/3 and w_2 = 2/3.

[a]the expected $Đ_2$ was calculated based on the experimental $Đ_1$ and $Đ_{12}$.

It is worth noting that the discrepancy between the theoretical and experimental $Đ_{12}$ can be attributed to a combination of factors such as dead chains from the first block, possible chain length-dependence of the deactivation rate, and the inaccuracies in measuring dispersity. Similarly, the presence of dead chains and inaccuracies of measurements lead to a higher calculated $Đ_{2.}$

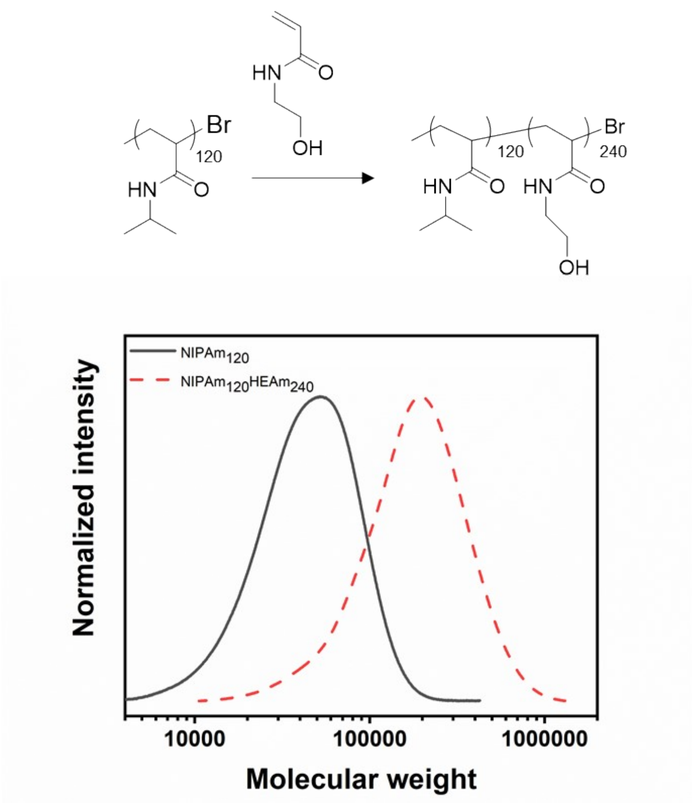

Figure S15. Synthesis of high-to-high dispersity PNIPAM-*block*-PHEAM via *in-situ* chain extension of PNIPAM (M_n = 34,200, $Đ$ = 1.53) with PHEAM to yield a final dispersity of 1.61 (M_n = 135,000).

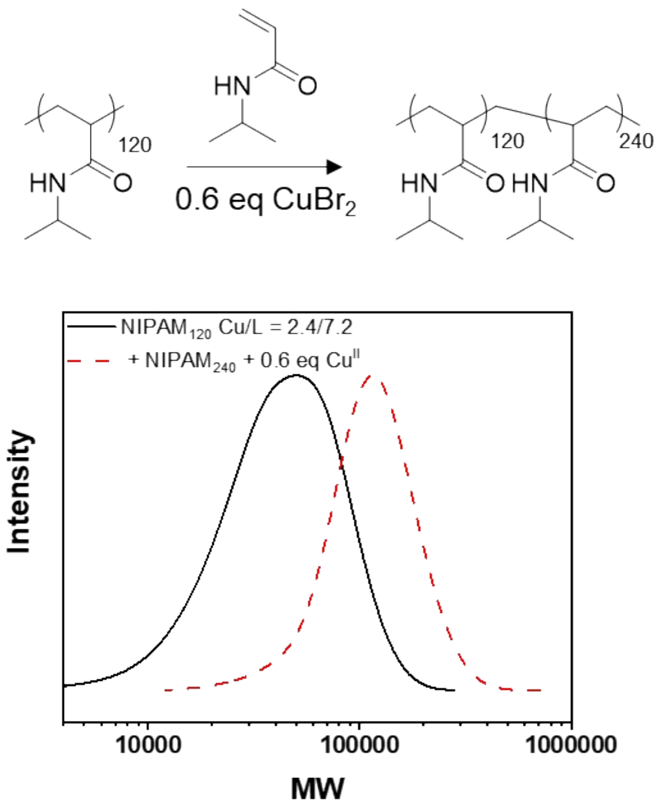

Figure S16. Synthesis of high-to-low dispersity PNIPAM-*block*-PNIPAM via in-situ chain extension of PNIPAM (M_n = 31,600, $Đ$ = 1.58) with PNIPAM to yield a final dispersity of 1.28 (M_n = 97,200).

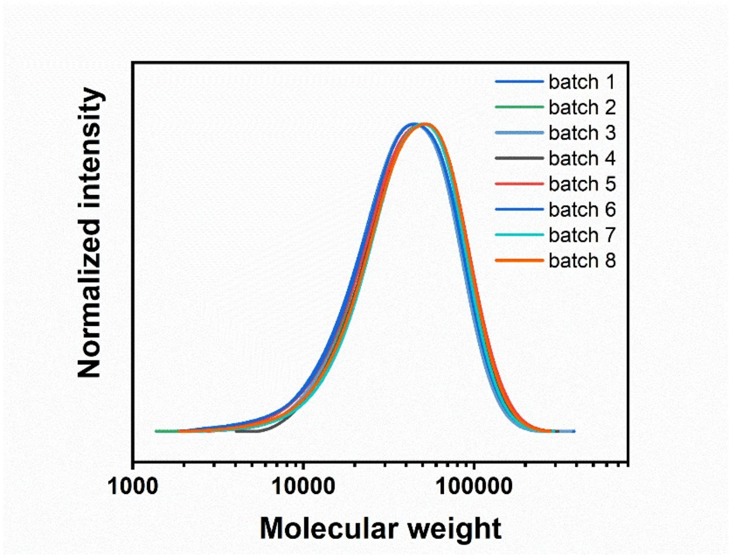

Figure S17. Reproducibility of high-dispersity PNIPAM batches (8 batches synthesized with conditions [NIPAM] : [GlyBiB] : [CuBr] : [Me_6Tren] = 120 : 1 : 2.4: 7.2)

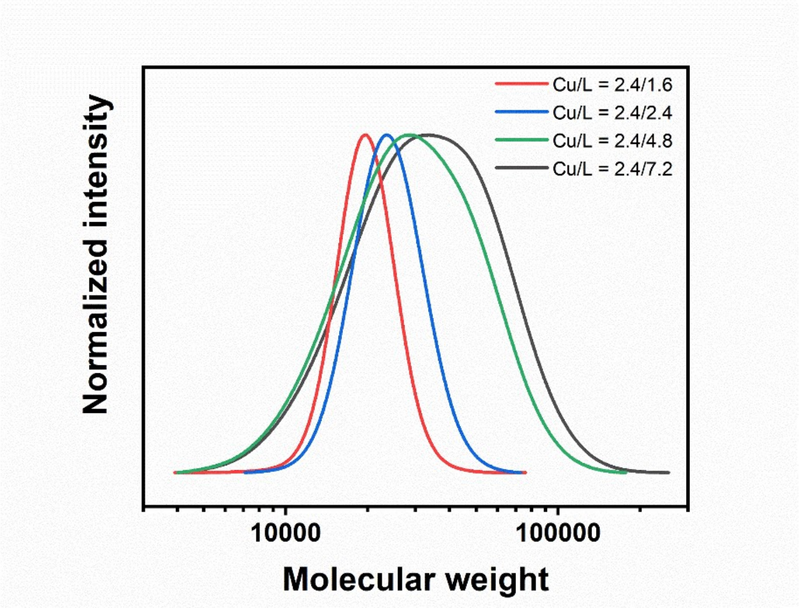

Figure S18. Tuning dispersity of PPEGA via varying the [CuBr] : [Me_6Tren] ratio.

Table S6. Tuning dispersity of PPEGA via varying the [CuBr] : [Me_6Tren] ratio.

Entry	Conversion (%)	[PEGA]:[GlyBiB] :[CuBr]:[Me_6Tren]	M_n^{SEC} (Da)	M_n^{theo} (Da)	$Đ$
1	92	[50]: [1]: [2.4] :[1.6]	18,900	22,100	1.07
2	99	[50]: [1]: [2.4] :[2.4]	22,600	23,800	1.10
3	96	[50]: [1]: [2.4] :[4.8]	24,100	23,000	1.39
4	99	[50]: [1]: [2.4] :[7.2]	26,500	23,800	1.44

Table S7. Polymerization of PPEGA with aligned M_p.

Entry	Conversion (%)	[PEGA]:[GlyBiB] :[CuBr]:[Me_6Tren]	M_n^{SEC} (Da)	M_n^{theo} (Da)	$Đ$
1	93	[91]: [1]: [2.4] :[1.6]	31,100	40,600	1.08
2	99	[50]: [1]: [2.4] :[7.2]	26,500	23,800	1.44

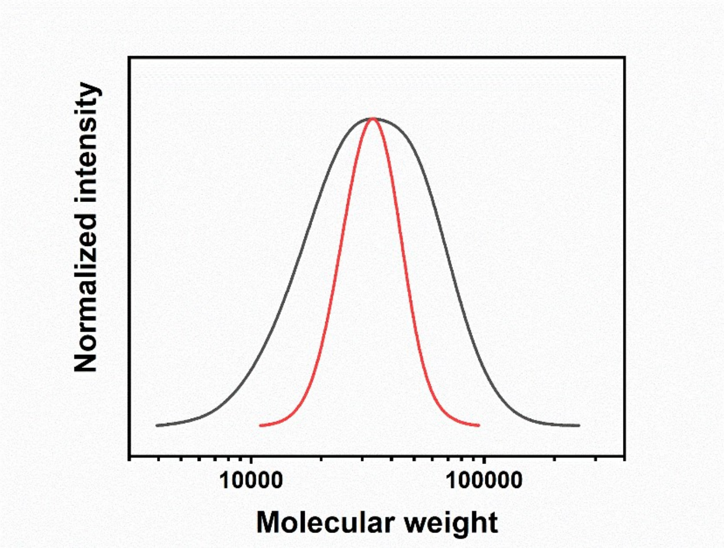

Figure S19. SEC traces of PPEGA with aligned M_p, Conditions are [PEGA] : [GlyBiB] : [CuBr] : [Me_6Tren] = [91]: [1]: [2.4] :[1.6] for the red trace and [50]: [1]: [2.4] :[7.2] for the black trace.

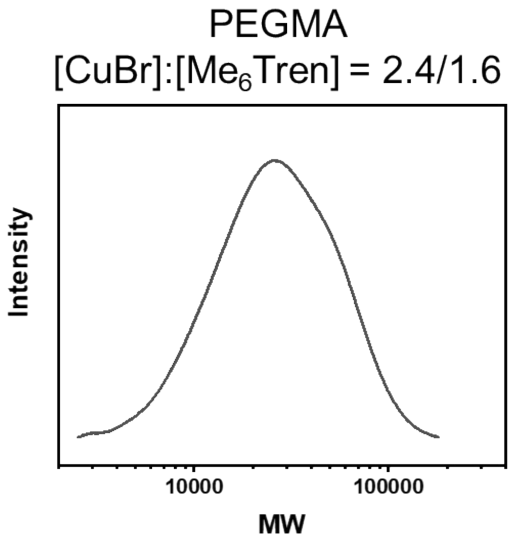

Figure S20. SEC traces of PPEGMA synthesized without excess ligand ([PEGMA] : [GlyBiB] : [CuBr] : [Me_6Tren] = [30]: [1]: [2.4] :[1.6]), M_n = 19,800, $Đ$ = 1.63.

Table S8. Synthesis of high-dispersity *in-situ* PNIPAM decablock copolymer

Block #	Target DP	Conversion (%)	M_n^{SEC} (Da)	M_n^{theo} (Da)	$Đ$
1	120	99	29,800	13,700	1.55
2	30	98	37,700	17,100	1.40
3	30	98	46,900	20,500	1.31
4	30	98	55,400	23,900	1.29
5	30	98	64,300	27,300	1.27
6	30	98	73,500	30,700	1.30
7	30	97	82,700	34,100	1.36
8	30	97	91,300	37,500	1.36
9	30	98	101,000	40,900	1.45
10	30	98	110,900	44,300	1.47

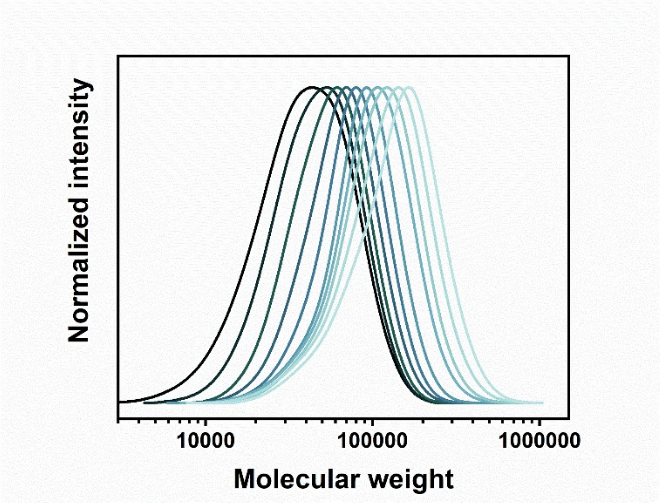

Figure S21. High-dispersity PNIPAM quasi-decablock synthesized via in-situ chain extensions (first block: [NIPAM] : [GlyBiB] : [CuBr] : [Me_6Tren] = [120] : [1] : [2.4]: [7.2])

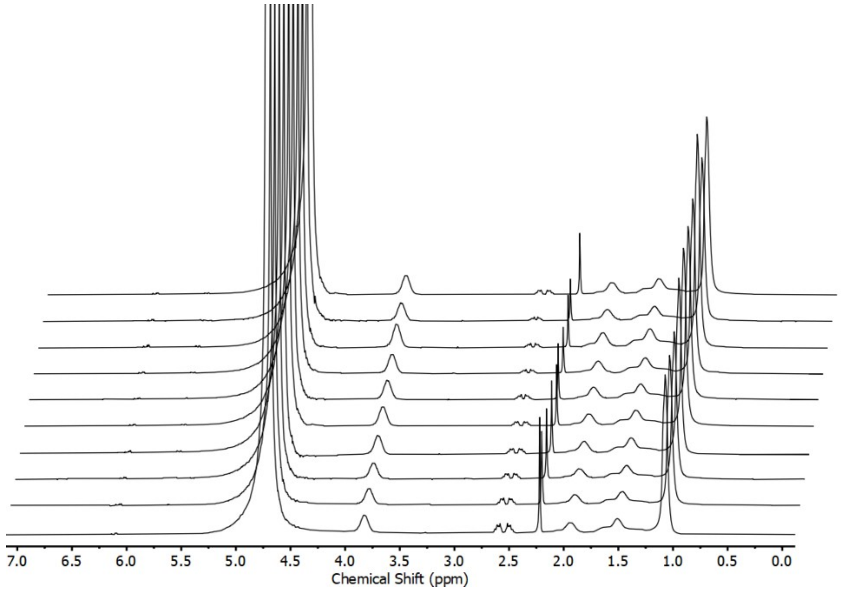

Figure S22. NMR spectra of block 1 (bottom spectrum) to block 10 (top spectrum) of the high dispersity PNIPAM quasi-decablock.(initial conditions for first block: [NIPAM] : [GlyBiB] : [CuBr] : [Me_6Tren] = 120 : 1 : 2.4: 7.2)

Table S9. Summary for the synthesis of PNIPAM-*block*-PHEAM-*block*-PNIPAM-*block*-PHEAM-*block*-PNAM pentablock copolymer.

Block	DP	Conversion (%)	M_n^{SEC} (Da)	M_n^{theo} (Da)	Đ
NIPAM	120	99	35,400	13,700	1.55
HEAm	120	98.5	83,300	27,400	1.46
NIPAM	120	95	132,200	40,500	1.41
HEAm	120	92	176,700	53,900	1.61
NAM	120	90	221,600	70,300	1.75

Table S10. Summary for the synthesis of in-situ PNIPAM-*block*-PHEAM-*block*-PNIPAM-*block*-PHEAM-*block*-PNAM pentablock copolymer without prior degassing and in the presence of ~6.5 ml headspace (8.5 ml of initial polymerization solution in 15 ml vial).

Block #	Monomer	Target DP	Conversion (%)	M_n^{SEC} (Da)	M_n^{theo} (Da)	Đ
1	NIPAM	120	99	42,000	13,700	1.36
2	HEAm	30	96	56,200	17,100	1.27
3	NIPAM	30	98	68,000	20,600	1.23
4	HEAm	30	95	82,600	24,000	1.30
5	NAM	30	98	95,000	28,300	1.36

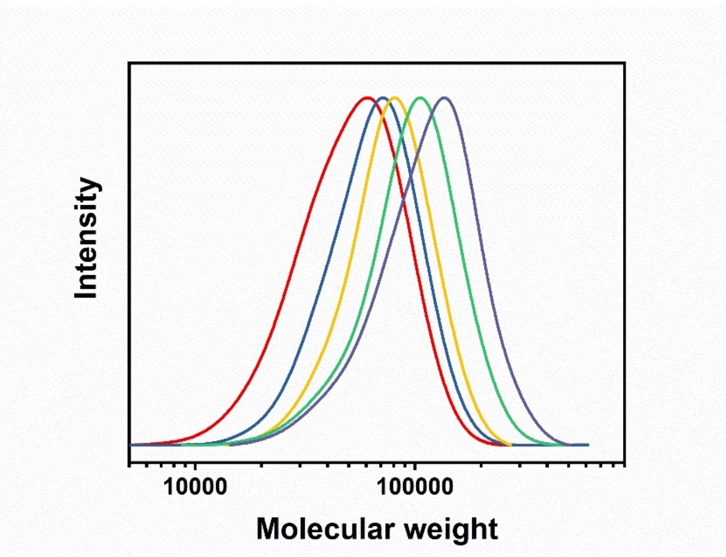

Figure S23. SEC traces of in-situ PNIPAM-*block*-PHEAM-*block*-PNIPAM-*block*-PHEAM-*block*-PNAM pentablock copolymer without prior degassing and in the presence of ~6.5 ml headspace (8.5 ml of initial polymerization solution in 15 ml vial).

Table S11. RAFT polymerization of PNIPAM (DP = 120) using 2-cyano-2-propyl dodecyltrithiocarbonate

Monomer	$[NIPAM]_0/[CTA]_0/[AIBN]_0$	Solvent	Conversion (%)	M_n^{th} (Da)	M_n^{SEC} (Da)	*Đ*
NIPAM	120/1/0.1	dioxane	72	10,100	14,500	1.08

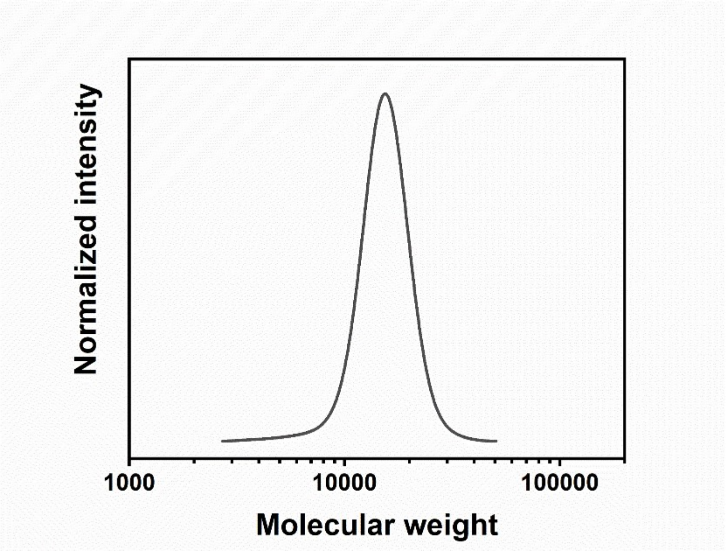

Figure S24. SEC trace of RAFT-PNIPAM. Experimental conditions outlined in Table S10.

Table S12. Photo-ATRP of methyl acrylate in DMSO using GlyBiB as the initiator

Monomer	$[MA]_0/[GlyBiB]_0/[CuBr_2]_0/[Me_6Tren]$	Solvent	Conversion (%)	M_n^{th} (Da)	M_n^{SEC} (Da)	*Đ*
Methyl acrylate	120/1/0.01/0.12	DMSO	93	9,800	11,200	1.06

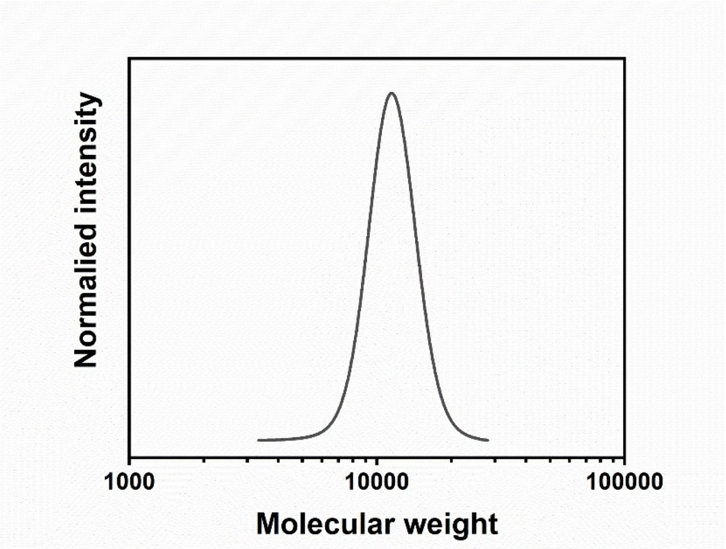

Figure S25. SEC trace of poly(methyl acrylate) synthesized via photo-ATRP in DMSO using GlyBiB as the initiator. Experimental conditions outlined in Table S11.
